# Enzymatic Hydrolysis of Bacterial Cellulose for the Production of Nanocrystals for the Food Packaging Industry

**DOI:** 10.3390/nano10040735

**Published:** 2020-04-11

**Authors:** Cesare Rovera, Filippo Fiori, Silvia Trabattoni, Diego Romano, Stefano Farris

**Affiliations:** 1 DeFENS, Department of Food, Environmental and Nutritional Sciences, University of Milan, via Celoria 2, 20133 Milan, Italy; cesare.rovera@unimi.it (C.R.); filippo.fiori@studenti.unimi.it (F.F.); diego.romano@unimi.it (D.R.); 2 Department of Materials Science, University of Milano Bicocca, via Cozzi 55, 20125 Milan, Italy; silvia.trabattoni@unimib.it; 3 INSTM, National Consortium of Materials Science and Technology, Local Unit University of Milan, via Celoria 2, 20133 Milan, Italy

**Keywords:** bacterial cellulose, cellulase, coating, endoglucanase, nanocomposite, oxygen barrier

## Abstract

Bacterial cellulose nanocrystals (BCNCs) obtained by enzymatic hydrolysis have been loaded in pullulan biopolymer for use as nanoparticles in the generation of high-oxygen barrier coatings intended for food packaging applications. Bacterial cellulose (BC) produced by *Komagataeibacter sucrofermentans* was hydrolyzed by two different enzymatic treatments, i.e., using endo-1,4-β-glucanases (EGs) from *Thermobifida halotolerans* and cellulase from *Trichoderma reesei*. The hydrolytic activity was compared by means of turbidity experiments over a period of 145 h, whereas BCNCs in their final state were compared, in terms of size and morphology, by atomic force microscopy (AFM) and dynamic light scattering (DLS). Though both treatments led to particles of similar size, a greater amount of nano-sized particles (≈250 nm) were observed in the system that also included cellulase enzymes. Unexpectedly, transmission electron microscopy (TEM) revealed that cellulose nanoparticles were round-shaped and made of 4–5 short (150–180 nm) piled whiskers. Pullulan/BCNCs nanocomposite coatings allowed an increase in the overall oxygen barrier performance, of more than two and one orders of magnitude (≈0.7 mL·m^−2^·24 h^−1^), of pure polyethylene terephthalate (PET) (≈120 mL·m^−2^·24 h^−1^) as well as pullulan/coated PET (≈6 mL·m^−2^·24 h^−1^), with no significant difference between treatments (hydrolysis mediated by EGs or with the addition of cellulase). BCNCs obtained by enzymatic hydrolysis have the potential to generate high oxygen barrier coatings for the food packaging industry.

## 1. Introduction

Cellulose synthesized by plants or bacteria is an almost inexhaustible organic polymer resource on Earth and has global economic importance [1]. Currently, bacterial cellulose (BC), produced by some acetic acid bacteria, is widely recognized as a multi-functional nano-biomaterial. BC is composed of linear glucan molecules, joined through hydrogen bonds, very similar in appearance to those found in plants. BC is produced by different bacteria, belonging to the *Acetobacteriaceae*, *Rhizobiaceae, Alcaligenaceae*, *Pseudomonadaceae*, *Enterobacteriaceae*, and *Clostridiaceae* families. The main strains used at the industrial level are *Komagataeibacter xylinus*, *Gluconacetobacter hansenii*, *Acetobacter pasteurianus* and *Komagataeibacter sucrofermentans*, which typically synthesizes BC of a molecular chain length ranging from 1 to 9 µm [2], at a rate of approximately 200,000 glucose molecules per second [3]. Unlike plant cellulose, bacterial cellulose produced by certain microorganisms, originates via a unique mechanism in the synthesis of chain molecules, followed by a subtle self-assembly process. However, when compared with other conventional natural or synthesized counterparts, BC performs better in areas such as biomedicine, functional devices, water treatment, nanofillers, etc. for its distinctly superior chemical purity, crystallinity, biocompatibility, and ultrafine network architecture [4]. Cellulose produced by bacteria is devoid of other molecules, such as lignin, hemicellulose and pectin, and further, the spatial arrangement of the pre-microfibril aggregation provides a high crystallinity, up to 84–89% [5], although the data for plant cellulose varies from 40% to 60% [6]. Otherwise, BC isolation and purification are relatively simple, not requiring energy- or chemical-intensive processes [7].

Cellulose nanocrystals (CNCs) are probably the most intriguing derivative of cellulose for a broad range of applications, including medical devices, purification/cleaning systems (e.g., membranes), displays, green building materials (e.g., insulating panels), and innovative packaging materials. CNCs are obtained through top-down processes (e.g., chemical, physical, and mechanical processes), which aim to isolate the crystalline phase from the amorphous regions of the parental, macro-sized cellulose fibers; the process eventually results in nano-objects characterized by high tensile strength, high length–diameter ratio, high specific surface area, and biodegradability [8]. For these reasons, CNCs represent an excellent alternative to the inorganic filler used in polymeric materials for the generation of nanocomposites.

The most commonly used method to obtain nanocrystals is acid hydrolysis (e.g., using sulfuric and hydrochloric acid), which promotes removal of the amorphous domains that are regularly distributed along the microfibers, and leads to the formation of rod-like cellulose nanocrystals [9]. However, the preparation of CNCs by concentrated acid solution is energy-consuming and environmentally hazardous. In addition, an overall decrease in the mechanical performance of nanocrystals obtained by acid hydrolysis has been reported [10]. For these reasons, finding more efficient top-down approaches with less environmental impact to obtain CNCs is a high priority.

Enzymatic hydrolysis has the potential to achieve higher yields, higher selectivity, lower energy costs, and milder operating conditions than chemical processes employed in the conversion of cellulosic biomass into nanocrystals. However, some technical challenges remain to be addressed for the full exploitation of the enzymatic hydrolysis. In particular, enhancing the efficiency of enzymes (in terms of enzymatic activity, U/mg), extending the pH and temperature operating window and improving the selectivity toward the amorphous or the crystalline regions of the cellulose substrate are of utmost importance. More specifically, as far as the selectivity target is concerned, cellulase enzymes are not selective, as cellulose is a mixture of endo-1,4-β-glucanases (EGs), exoglucanases or exocellobiohydrolases (CBHs), and β-glucosidases (BGLs), each of which performs a specific hydrolytic action [11].

In our previous work, we demonstrated that cellulases facilitate the obtaining of bacterial cellulose nanocrystals (BCNCs), with the best outcome (in terms of morphology and size distribution) obtained using a high amount of enzymes [12]. However, the overall yield of the process was very low (≈5%), which was ascribed to the high intensity of EGs’ and CBHs’ synergistic action, up to the obtainment of cellobiose and glucose. 

With the goal of rendering the overall BC hydrolysis process more selective toward the amorphous regions, thereby increasing the overall yield of nanocrystals, in this work we decided to carry out the enzymatic hydrolysis of BC using only EGs. The hydrolytic activity of EGs was compared to that of cellulases, through dimensional analysis of the obtained BCNCs and quantification of the reducing sugars at the end of the process. Finally, BCNCs were used for the generation of oxygen barrier bionanocomposite coatings, deposited on polyethylene terephthalate (PET) for the food packaging industry.

## 2. Materials and Methods 

### 2.1. Prodution of Macro-Sized Bacterial Cellulose (BC)

The production of BC followed the same procedure used by Rovera et al. (2016) [12]. Briefly, BC was produced by static fermentation (seven days at 30 °C in a Hestrin and Schramm culture medium) using *Komagataeibacter sucrofermentans* DSM 15973 strain (Leibniz Institute DSMZ-German Collection of Microorganisms and Cell Cultures, Braunschweig Germany). BC pellicles were washed with deionized water and boiled in an alkaline solution (NaOH 1M) for 30 min to remove the residual bacterial cells. After washing several times with distilled water, the cellulosic material was homogenized for 15 min with an Ultra-turrax^®^ T25 Basic homogenizer with an S25 N-18 G dispersing tool (Ika-Werke, Stanfen, Germany) at 12,000 rpm and finally freeze-dried at −55 °C and 0.63 mbar for 24 h using an ALPHA 1-2 LDplus freeze dryer (Martin Christ, Osterode am Harz, Germany).

### 2.2. Enzymatic Hydrolysis of BC

The hydrolysis of BC was performed using two different enzymes: an endo-1,4-β-glucanase from *Thermobifida halotolerans* in concentrated (≈150 U/mL) liquid form (Megazyme, specific enzymatic activity ≈26.8 U/mg protein at pH 8.5 and 60 °C, Astori Tecnica, Poncarale, Italy) and a cellulase from *Trichoderma reesei* in powder form (ATCC26921, specific enzymatic activity ≈6.5 U/mg protein at pH 5 and 55 °C, Sigma-Aldrich, Milano, Italy).

In both cases, a stock of BC in water was prepared by adding 12 g of freeze-dried BC to 88 g of distilled water. Then, 1 g of the dispersion was added to 30 g of buffer solution, specifically, a Tris buffer solution (50 mM, pH 8.5), containing 1 mg/mL BSA, was used for the endo-1,4-β-glucanase-mediated hydrolysis, while a sodium acetate buffer solution (0.1 M, pH 5) was used for the cellulase-mediated hydrolysis. Before the addition of the enzymes, BC dispersions were homogenized using an Ultra-turrax^®^ T25 Basic homogenizer with an S25 N-18 G dispersing tool (Ika-Werke, Stanfen, Germany) at 8000 rpm for 5 min. The dispersions were then kept overnight in an incubator at 60 ± 1 °C and 55 ± 1 °C for the endo-1,4-β-glucanase- and cellulase-containing dispersions, respectively, intended to promote the diffusion of water molecules into the cellulosic network. The enzymatic hydrolysis of BC, irrespective of the enzyme used, was conducted for a minimum of 2 h and a maximum of 145 h, according to the formulations reported in Table 1.

### 2.3. Pullulan/Bacterial Cellulose Nanocrystals (BCNCs) Nanocomposite Coating Preparation

Pullulan powder (PI-20 grade, M_w_ ≈ 200 KDa), purchased from Hayashibara Biochemical Laboratories Inc. (Okayama, Japan) was used as biopolymer phase. Pullulan powder (10 wt%) and BCNCs (10 wt%, wet basis, i.e., a water solution with a BCNC concentration of 1.51 wt%, so that the dry amount of BCNCs in the final water-based dispersion was 0.151 wt%) were mixed together with water at 25 °C for 30 min. under gentle stirring (800 rpm). The pullulan/BCNCs water dispersion was laid on a polyethylene terephthalate (PET) plastic substrate (AryaPET-A410, 12.0 ± 0.5 µm thick, JBF RAK LLC, Ras Al Khaimah, United Arab Emirates), kindly provided by SAES Coated Films S.p.A. (Roncello, Italy). To this scope, 3 mL of the coating dispersion were poured onto the corona-treated side of a rectangular (24 × 18 cm^2^) PET specimen, previously placed on an automatic film applicator (TQC Sheen Instruments, Capelle aan den IJssel, the Netherlands). The coating process was carried out according to the ASTM D823-07-Practice C, at a constant speed of 150 mm·min^−1^, using a horizontal steel rod with an engraved pattern, which yielded final coatings of comparable nominal dry thickness of ≈1 µm. Water evaporation was achieved using a constant and perpendicular flux of mild air (25.0 ± 0.3 °C for 2 min) at a distance of 40 cm from the applicator, as reported by Unalan et al., 2016 [13]. Coated films were then stored at 23.0 ± 0.5 °C in a desiccator for 1 week before characterization.

### 2.4. Analyses

The evolution of the enzymatic hydrolysis was monitored at 3 h intervals in turbidity experiments, over a temporal window of 145 h, according to the procedure reported in our previous work [12]. Spectrophotometric measurements were performed using a Lambda 25 spectrophotometer (PerkinElmer, Waltham, MA, USA) at wavelengths between 380 and 800 nm in transmittance mode. For all samples, the area under the transmittance spectrum was calculated by means of PerkinElmer UV WinLab software (version 6.0.4.0738) and plotted over time. After turbidimetric analysis came a process of removing macroscopic aggregates of hydrolyzed cellulose, possibly present in the final water solutions, that relied on a Buchner filtration system using filter paper (grade 4/N, particles retention: 2 µm, Munktell, Sweden); this removal was performed in all mixtures.

Atomic force microscopy (AFM) experiments were performed using a Nanoscope V Multimode (Bruker, Karlsruhe, Germany) in intermittent-contact mode after dropping 10 μL of 1:10 diluted BCNC dispersion onto a mica substrate. The images were collected at a resolution of 512 × 512 pixels using silicon tips (force constant, 3 N/m; resonance frequency, ≈75 kHz). Dimensional calculations for the acquired images were conducted with Nanoscope software (version 7.30; Bruker, Karlsruhe, Germany).

A transmission electron microscope (TEM) was used to capture images of the BCNCs (a LEO 912 AB energy-filtering transmission electron microscope operating at 80 kV; Zeiss, Oberkochen, Germany). Digital images were recorded with a ProScan 1K Slow-Scan CCD camera (ProScan, Scheuring, Germany). Samples for the TEM analyses were prepared, according to the negative staining technique, by drop-casting a few microliters of dispersion onto a glow-discharged Formvar-coated Cu grid (400-mesh) and letting the samples rest for 1−2 min, then blotting away the excess of suspension and contrasting with uranyl acetate. 

Information on the size distribution of BCNCs was obtained through photon correlation spectroscopy, using a dynamic light scattering (DLS) Nanotrac Flex in-situ analyzer (Microtrac Inc., USA). Analyses were carried out at 25 °C, with a stabilization time of 60 s, and using water’s viscosity (v = 0.8872 cP) and refractive index (n = 1.330) values. The software used the nonnegative least-squares algorithm to calculate the size distribution.

Thermogravimetric analysis (TGA) was carried out using a Mettler Toledo SDTA 851^e^ (Mettler Toledo, Greifensee, Switzerland). Filtered water solutions (≈5 mg) were placed in alumina pans (70 µL) and initially kept at 25 °C for 10 min, after which they were heated up to 220 °C at a rate of 5 °C·min^−1^ in inert (50 mL·min^−1^ N_2_) environment.

Cellobiose and glucose were quantified by high-performance liquid chromatography (HPLC), using a Merck Hitachi L-7100 system with an Aminex HPX-87P column (300 mm × 7.8 mm; Biorad Laboratories, Hercules, CA, USA) and an evaporative light-scattering detector (Sedex 75; Sedere, Alfortville, France; conditions: air 3.5 bar, 50 °C). The analysis was carried out at 60 °C, using water (0.5 mL/min) as eluent.

The oxygen barrier performance of the bionanocomposite-coated PET films were assessed using a TotalPerm permeability analyzer (Permtech Srl, Lucca, Italy) equipped with an electrochemical sensor. The oxygen transmission rate (OTR, mL·m^−2^·24 h^−1^) of coated films was determined according to the ASTM standard method F2622-08, with a carrier flow (N_2_) of 10 mL·min^−1^ at 23 °C, 0% relative humidity (RH) and 80% RH, at 1 atmosphere oxygen partial pressure difference on the two sides of the specimen. Analyses were carried out with the coated side of each sample facing the upper semi-chamber, into which the humid test gas (oxygen) was flushed. The final values result from three replicate measurements.

### 2.5. Statistical Analysis

Data were analyzed using Statgraphics Plus 4.0 software (STSC, Rockville, MD, USA), and one-way analysis of variance was used to check for differences between samples. The significance level (*p*) was fixed at 0.05.

## 3. Results and Discussion 

### 3.1. Kinetics of the Enzymatic Hydrolysis

**Figure 1:**
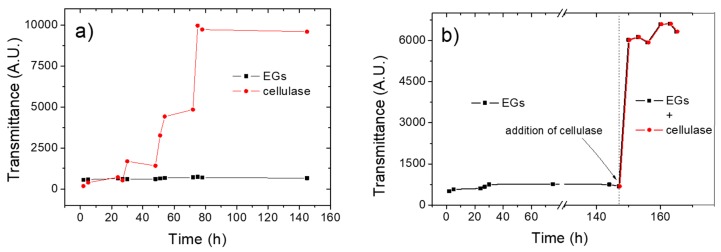
(**a**) Evolution of transmittance (expressed as the area under each spectrum) during 145 h of hydrolysis conducted using endo-1,4-β-glucanases (EGs) or cellulase enzymes; (**b**) Evolution of transmittance (expressed as the area under each spectrum) during 165 h of hydrolysis conducted using EGs (up to 145 h) and then adding cellulase.

Turbidity experiments via spectrophotometric measurements represent a viable approach to monitor the evolution of the enzyme-mediated hydrolysis of cellulose, the basic principle being that a decrease in turbidity (i.e., an increased transmittance) is expected for extended hydrolysis times, because of the reduced size of the BC fibrils [12]. Figure 1a shows the hydrolysis kinetics, expressed as transmittance evolution over time, for both the endo-1,4-β-glucanase- and the cellulase-containing systems. 

The disparate performances of the two enzymes is clearly demonstrated by the different pattern reflected in the ‘transmittance vs time’ plots. In particular, an almost straight line represents the evolution of the transmittance in the case of the hydrolysis mediated by the endo-1,4-β-glucanase, with only a subtle climbing phase observable after approximately after 50 h of hydrolysis. This suggests that this enzyme, in isolation, was virtually ineffective to promote a reduction in size of the parental macro-sized BC. By contrast, the plot obtained for the hydrolytic activity of cellulases confirmed previous observations, i.e., an initial lag phase followed by a rising tract after ≈50 h, which stopped when a steady state was reached after approximately 75 h [12]. The disparate activity of the two enzymes was confirmed by HPLC analysis of the hydrolyzed samples. Cellobiose and glucose were both found at the end of the hydrolysis process that involved cellulases (1.54 ± 0.04 g/L and 2.25 ± 0.12 g/L, respectively), whereas only cellobiose (1.36 ± 0.11 g/L) was found when EGs alone were used, thereby confirming the absence of the BGL enzymes, which specifically hydrolyze cellobiose into glucose units. Interestingly, a decreasing trend in transmittance was observed for both enzymes after approximately 75 h, i.e., an increase in turbidity occurred, probably due to the reaggregation of nanocrystals or micron-sized cellulose fibrils caused by the extensive hydrogen bonding between the chemically unmodified (i.e., –OH-rich) cellulose molecules. 

To corroborate the synergistic effect of EGs, CBHs, and BGLs in the hydrolysis of BC, we decided to combine the two processes described above. More specifically, the cellulase enzymes were added (500 μL solution, i.e., 16.25 U/mg) after 147 h of hydrolysis mediated by the endo-1,4-β-glucanase. As shown in Figure 1b, the addition of cellulases boosted the hydrolytic reaction insofar as a steep increase in the transmittance was observed soon after the addition of the enzymes (i.e., after 147 h). This synergistic effect was also observed macroscopically. In fact, visual inspection revealed a more homogeneous dispersion when cellulase enzymes were added to the system, relative to the dispersion obtained after the hydrolysis of BC in the presence of the endo-1,4-β-glucanase alone. At the same time, some macroscopic residues of untreated BC were also detected, probably because of the previously added EGs that might compete with cellulases for the binding sites on the cellulose backbone.

### 3.2. Morphology and Size Distribution of Hydrolyzed BC

**Figure 2:**
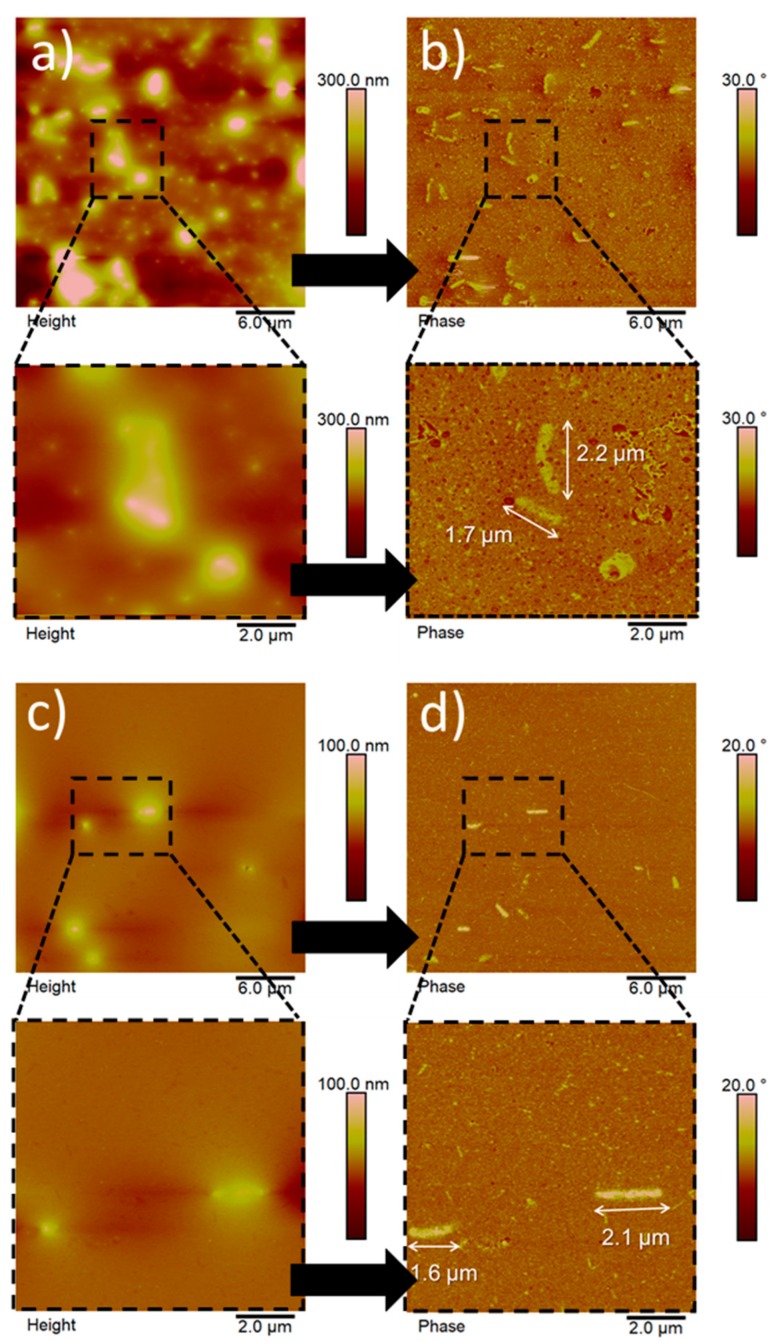
Atomic force microscopy (AFM) images of BC particles after hydrolysis mediated by EGs (panels **a**,**b**) and EGs + cellulase (panels **c**,**d**). Height images and corresponding phase contrast images are shown in the left and right column, respectively. Zoomed areas are displayed below the main panels (**a**–**d**).

Figure 2 displays the AFM images of nanoparticles obtained from the enzymatic hydrolysis of BC, reporting both the height and phase contrast signals (Figure 2a–d, respectively). 

To remove particles bigger than ≈2 µm, possibly present in the dispersion at the end of the process, a filtration step was carried out before acquisition of the images (see the experimental section). Regardless of the type of enzyme used, the final outcome was represented by rod-like, micron-sized particles and ellipsoidal/spherical particles of nanometer size. These particle shapes can be clearly identified in contrast phase images (Figure 2b,d). The phase contrast signal, arising from the power dissipated by the tip, is sensitive to the material stiffness, allowing the easier detection of nanosized structures that could be difficult to recognize in the corresponding height images [14]. These shapes differ greatly from the typical needle-like shape of BCNCs obtained by both acid hydrolysis [15] and enzymatic hydrolysis [12]. Although not common, the rod-like and ellipsoidal/spherical shapes have already been described in the context of cellulose nanoparticles. For example, Hafemann et al. reported on both rod-like and spherical shapes for CNCs obtained from royal palm tree agroindustrial waste [16]. In particular, they noted that rod-shaped particles were not uniform in size because of the intensity of the acid treatment. More specifically, these particles were degraded differently by the acid hydrolysis, to the extent that a shift from rod-shaped particles to spherical particles occurred under extended hydrolysis conditions. In our study, we made similar observations. It is thus plausible that rod-shaped particles represent less degraded cellulose, whereas smaller particles refer to nano-domains that underwent extensive enzymatic hydrolysis. 

The main difference between the two hydrolysis processes used in this study (i.e., in the presence of EGs or in the presence of both EGs and cellulose enzymes), relates to the size distribution of the particles, rather than to the absolute size and morphology of the particles. The enzymatic hydrolysis mediated by EGs (Figure 2a,b) yielded big particles of ≈1.8–2.0 µm in length and ≈300–350 nm in width together with smaller (in the nanometer range) particles. The same outcome was observed for the particles arising from the use of EGs, followed by the addition (after 147 h) of cellulase (Figure 2 c,d). Moreover, the particles’ thickness was lower when BC was treated with EG in combination with cellulases than in the presence of only EGs, which is consistent with the previously-described peripheral attack of the enzyme [12]. However, the number of big (micron-sized) particles was much higher for the enzymatic hydrolysis mediated by EGs, whereas the combined (i.e., EGs + cellulases) hydrolytic process led to a higher number of nanoparticles. 

**Figure 3:**
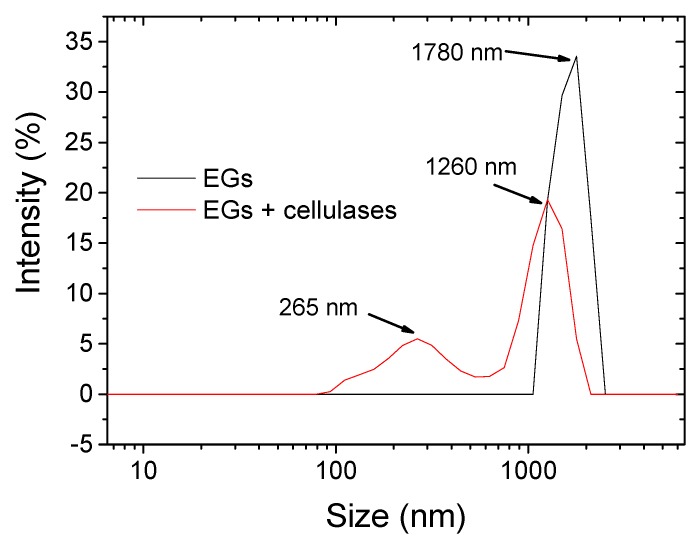
Size distribution intensity obtained by dynamic light scattering (DLS) measurements of hydrolyzed BC using endo-1,4-β-glucanases (EGs) and EGs + cellulase enzymes.

This finding was confirmed quantitatively by dynamic light scattering measurements. As shown in Figure 3, hydrolysis by EGs yielded a main family distribution centered at ≈1.8 µm, whereas the hydrolysis with EGs and cellulases led to two families of nanometric (≈250 nm) and micrometric (≈1.2 µm) size. 

These absolute values must be taken with caution, because DLS is not a suitable technique in determining the absolute size distribution of nonspherical particles. Rather, DLS is a useful tool to assess relative differences between particles that underwent the same process/treatment, as in this study [12]. Another confirmation of the greater amount of nano-sized particles for the BC treated with EGs and cellulase enzymes was indirectly provided by quantification of the solids content of the hydrolyzed water dispersions after filtration (see experimental part). As shown by thermogravimetric analysis (TGA) (Figure S1), the solids content at the end of the process amounted to ≈1.5 wt% and ≈2.3 wt% for the EGs-treated and EGs + cellulase-treated samples, respectively. This difference can be explained in terms of a higher amount of residual BC (micron-sized BC) in the filter paper when the EGs alone were used. 

**Figure 4:**
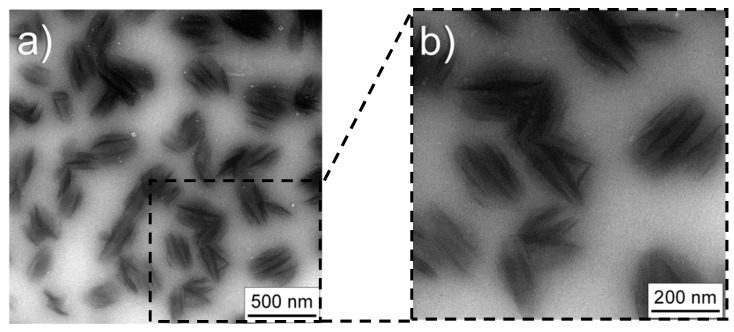
Transmission electron microscopy (TEM) images of bacterial cellulose nanocrystals after 145 h enzymatic hydrolysis using EGs and cellulase enzymes. Panel (**b**) is a zoomed area of panel (**a**).

As far as BCNCs are concerned, we used TEM to gather exhaustive information on the morphology and structure of these particles (Figure 4). 

TEM images of BCNCs obtained from the hydrolysis mediated by EGs and cellulases confirmed the prevalent spherical shape of nanoparticles, with an average diameter of approximately 200 nm (Figure 4a). At higher magnification, it was possible to observe that each nanoparticle did indeed seem built by 5–6 short (150–180 nm) crystals stacked on top of each other, all of them immersed in a less dense matrix (Figure 4b). This scenario suggests that each round nanoparticle is made of short crystalline regions, originally part of a fibril that was degraded by the enzyme. The light shadow that interpenetrates the space between the crystals may be due to low molecular weight products of the hydrolysis, such as short cellulose chains or even cellobiose and glucose. This explanation would align with the model of Martinez-Sanz et al. [17], according to which BC can be viewed as being composed of a core of solvent-impermeable crystallites and an outer region of amorphous cellulose.

### 3.3. Oxygen Barrier Properties

Although bacterial cellulose nanocrystals have demonstrated their potential for a variety of interesting applications in the food packaging sector, only a few of these have, to date, been explored, because the high cost of BC production is usually considered as the main limiting factor [18]. The most promising applications concern the use of BCNCs as organic filler to improve the ultimate mechanical and gas barrier properties of the packaging materials. In this work, we used BCNCs as an organic filler loaded in the biopolymer pullulan, a non-ionic exopolysaccharide obtained from the fungus-like yeast *Aureobasidium pullulans*. Because of its unique physico-chemical properties, pullulan has been widely used for the generation of high-performance packaging materials, especially in the form of oxygen barrier coatings [19]. To improve the oxygen barrier performance of pullulan-based coatings, several inorganic and organic fillers have traditionally been used, such as microfibrillated cellulose [20,21], silica nanoparticles [22], graphene oxide [13,23], and mica [24]. In this work, we used, for the first time, BCNCs for the development of pullulan bionanocomposite coatings with high oxygen barrier properties. 

Table 2 displays the OTR values of bare PET, pullulan-coated PET, and PET coated with pullulan bionanocomposites, obtained using BCNCs from both EGs and EGs + cellulose enzymatic treatments.

The plastic substrate exhibits moderate oxygen barrier properties (≈120 mL·m^−2^·24 h^−1^), which greatly improved after the deposition of the pristine pullulan coating (95% reduction). The oxygen barrier performance improved even further for the pullulan/BCNCs coatings, with an overall decrease over the pure PET of more than two orders of magnitude. This outstanding performance has been already observed for ‘nano’ systems, whereby both interfacial effects and an increased tortuous path play a crucial role. In the case of BCNCs, we are inclined to consider the interface effects predominant over the tortuous path phenomenon, because of the lower surface area of 1D nanoparticles (as BCNCs), relative to 2D nanoparticles (e.g., platy nanofillers such as graphene and montmorillonite), which eventually makes BCNCs less efficient as physical impedance to the permeation of oxygen.

Experimental data seemed to indicate a better performance for the BCNCs obtained using both enzymes (EGs and cellulose), but lack of statistical difference does not allow us to draw any significant conclusion on the best performing nanocomposite coating. Both bionanocomposite coatings exhibited a very high oxygen barrier performance (OTR < 1 mL·m^−2^·24 h^−1^) under dry conditions, making the final material as good as commercially available solutions (e.g., PET coated with ethylene(vinyl alcohol), poly(vinyl alcohol), and polyvinylidene chloride at 0% RH. This performance was almost completely lost at 80% RH. This behavior has been extensively explained, in terms of water molecules’ competing for the polar groups (predominantly hydroxyl groups) displaced along both the biopolymer and filler backbones and taking part in intermolecular hydrogen bonding [13]. The negative impact of water molecules is so obvious that, at high RHs, the OTR value of pure PET was almost restored (Table 2).

## 4. Conclusions

Enzymatic hydrolysis is an alternative approach to most common routes (chemical and/or physical routes) toward the reduction in size of bacterial cellulose, featuring unique performance in terms of environmental impacts and process conditions. However, the non-selective attack of cellulase enzymes, while a big advantage when glucose is the target (e.g., biofuel production), is a limitation for the manufacturing of BCNCs, due to the extensive degradation it produces of both amorphous and crystalline regions. Using EGs alone allowed for the achievement of a less extensive degradation of the cellulose backbone (as demonstrated by the absence of glucose after hydrolysis), which was, however, reflected in a less effective reduction in the size of the macroscopic BC (as shown by AFM analyses). EGs-treated BC indeed yielded both macro-particles and micro-particles, with the smallest particles (i.e., <2 μm) accounting for only ≈1.5 wt% of the total (as demonstrated by the TGA experiments). If new enzymatic routes are found to the obtainment of BCNCs at high yields, then these nanofillers can emerge as a green solution for the design of high-performing materials, with potential uses in different fields, e.g., food packaging.

## Figures and Tables

**Table 1:** Cellulase/bacterial cellulose (BC) and endo-1,4-β-glucanases/BC ratios with the specific amount of enzyme and enzymatic units.

**Cellulase ^a^/BC ^b^ (w/w)**	**Cellulase ^a^ (mg)**	**Enzyme Unit (U)**
1:4	2.5	16.25
**Endo-1,4-β-glucanase ^c^/BC ^b^ (v/w)**	**Endo-1,4-β-glucanase ^c^ (μL)**	**Enzyme Unit (U)**
1:4	600	90

^a^ Prepared as a stock solution of the enzyme in water (5 mg/mL). ^b^ Prepared from a starting 12 wt% aqueous dispersion. ^c^ Prepared from the suspension as received.

**Table 2:** Oxygen transmission rate (OTR) values at 0% relative humidity (RH) and 80% RH for bare polyethylene terephthalate (PET), pullulan-coated PET, and PET coated with pullulan/BCNCs bionanocomposite coatings.

Filler Content	OTR (0% RH) (mL·m^−2^·24 h^−1^)	OTR (80% RH) (mL·m^−2^·24 h^−1^)
PET	120.3 ± 2.1 ^a^	109.5 ± 1.7 ^A^
PET/pullulan	6.2 ± 0.8 ^b^	100.73 ± 3.23 ^A^
PET/pullulan/BCNCs (EGs)	0.840 ± 0.12 ^c^	94.14 ± 2.19 ^B^
PET/pullulan/BCNCs (EGs + cellulase)	0.702 ± 0.09 ^c^	89.71 ± 2.21 ^B^

^a,b,c,A,B^ Different superscripts within a group (i.e., within each parameter) denote a statistically significant difference (*p* < 0.05). Error around the mean value represents the standard deviation.

## Supplementary Materials

The following are available online at https://www.mdpi.com/2079-4991/10/4/735/s1, Figure S1: Thermogravimetric analyses of BC hydrolyzed by EGs (a) and cellulase (b) after filtration.
