# Supplementary material for: Enzymatic Hydrolysis of Bacterial Cellulose for the Production of Nanocrystals for the Food Packaging Industry
Source: Nanomaterials (Basel). 2020 Apr 11;10(4):735. doi: 10.3390/nano10040735 (PMC7221805; doi:10.3390/nano10040735)
Supplement: Supplementary file 1 [file nanomaterials-10-00735-s001.pdf]

nanomaterials

Supplementary Materials

Enzymatic Hydrolysis of Bacterial Cellulose for the Production of Nanocrystals for the Food Packaging Industry

Cesare Rovera [1], Filippo Fiori [1], Silvia Trabattoni [2], Diego Romano [1,3], and Stefano Farris [1,3,*]

[1] DeFENS, Department of Food, Environmental and Nutritional Sciences, University of Milan, via Celoria 2, 20133 Milan, Italy; cesare.rovera@unimi.it (C.R.); filippo.fiori@studenti.unimi.it (F.F.); diego.romano@unimi.it (D.R.)
[2] Department of Materials Science, University of Milano Bicocca, via Cozzi 55, 20125 Milan, Italy; silvia.trabattoni@unimib.it
[3] INSTM, National Consortium of Materials Science and Technology, Local Unit University of Milan, via Celoria 2, 20133 Milan, Italy
* Correspondence: stefano.farris@unimi.it; Tel.: +39-25-031-6805

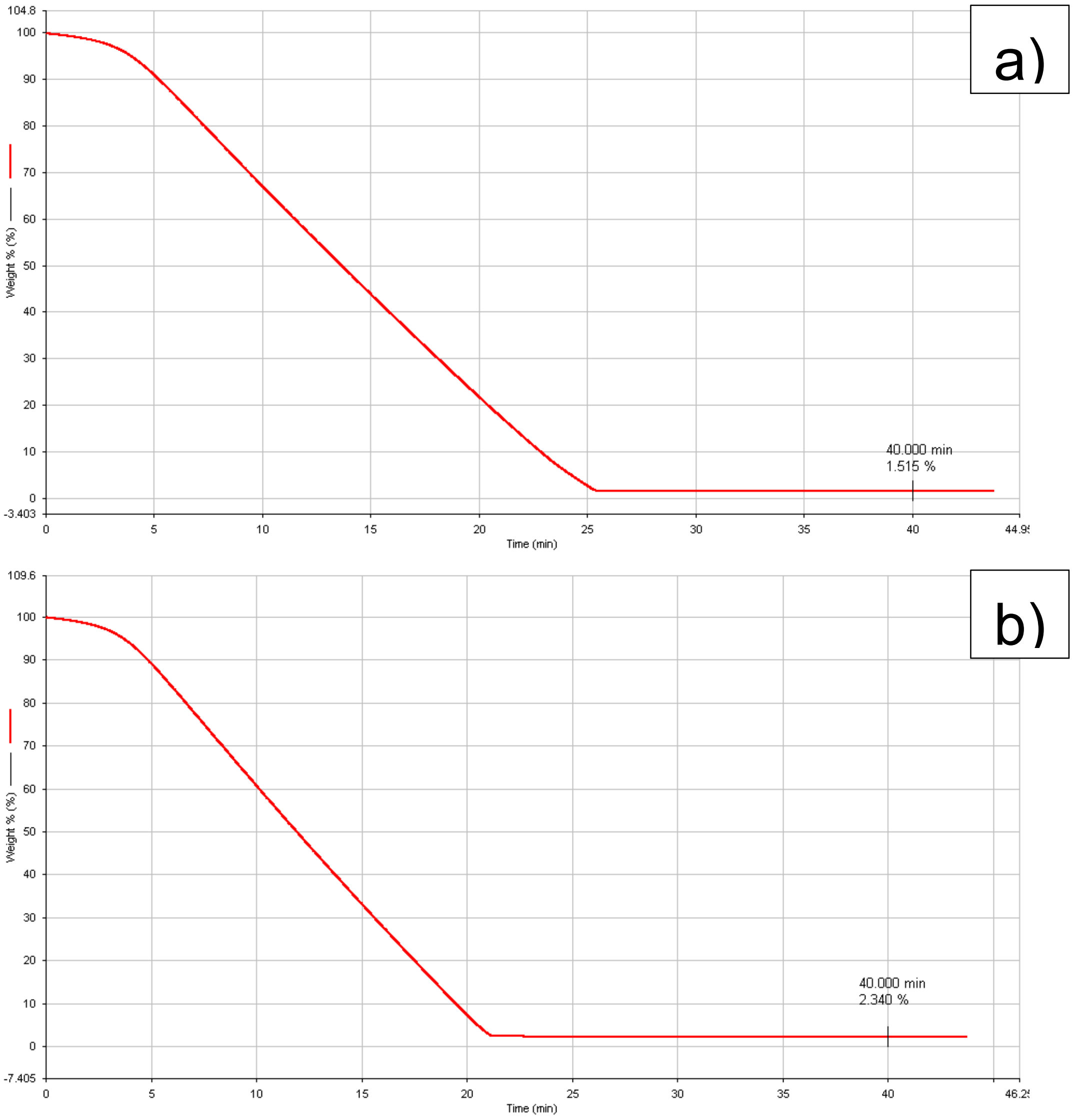

Figure S1. Thermogravimetric analyses of BC hydrolyzed by EGs (a) and cellulase (b) after filtration (see main text for details).

© 2020 by the authors. Submitted for possible open access publication under the terms and conditions of the Creative Commons Attribution (CC BY) license (http://creativecommons.org/licenses/by/4.0/).
